# The antibiotic resistance of *Helicobacter pylori* to five antibiotics and influencing factors in an area of China with a high risk of gastric cancer

**DOI:** 10.1186/s12866-019-1517-4

**Published:** 2019-07-04

**Authors:** Dan Wang, Qianqian Guo, Yuan Yuan, Yuehua Gong

**Affiliations:** 1 Tumor Etiology and Screening Department of Cancer Institute and General Surger, the First Hospital of China Medical University, Shenyang, 110001 China; 2 Key Laboratory of Cancer Etiology and Prevention in Liaoning Education Department, the First Hospital of China Medical University, Shenyang, 110001 China; 3 Key Laboratory of GI Cancer Etiology and Prevention in Liaoning Province, the First Hospital of China Medical University, Shenyang, 110001 China

**Keywords:** *H. pylori*, Antibiotic resistance, Virulence factors, High-risk area of gastric cancer

## Abstract

**Background:**

*H. pylori* exhibits antibiotic resistance with regional differences. In this paper, we explored antibiotic resistance of *H. pylori* to five antibiotics in an area with a high risk of gastric cancer.

**Results:**

*H. pylori* resistance rates to metronidazole, levofloxacin, clarithromycin, amoxicillin, and tetracycline were 78.0, 56.0, 31.0, 9.0, and 15.0%, respectively. Double, triple, quadruple, and quintuple resistance rates were 23, 20, 6, and 4%, respectively. The clarithromycin and multidrug resistance rates were significantly higher in males than females (clarithromycin: 44.4% vs 15.2%, respectively, *P* = 0.002; multidrug: 75.5% vs 37.2%, respectively; *P* < 0.001). During the three periods of 1998–1999, 2002–2004 and 2016–2017, the resistance rates to levofloxacin and amoxicillin were increasing (OR: 2.089, 95%CI: 1.142–3.821, *P* = 0.017; and OR: 5.035, 95%CI: 1.327–19.105, *P* = 0.018, respectively). The antibiotic resistance rates were unassociated with the host disease state. Metronidazole resistance was lower in the *vacA*s1m1/m2 group than the *vacA*s1m1m2 group (65% vs 85.7%, respectively; *P* = 0.026). As for levofloxacin resistance, it was higher with *cagA* ^+^ than *cagA*^−^ (60.9% vs 23.1%, respectively; *P* = 0.020) but lower with *slyD*^+^ than *slyD*^−^ (41.4% vs 68.5%, respectively; *P* = 0.009). Clarithromycin had a lower resistance rate with *iceA*^++^ than *iceA*^−+^ (19.7% vs 52.4%, respectively; *P* = 0.017). For amoxicillin, the *iceA*^++^ group had a lower resistance rate than the *iceA*^−−^ group (1.6% vs 27.8%, respectively; *P* = 0.009).

**Conclusions:**

The total resistance rates of *H. pylori* to metronidazole, levofloxacin, clarithromycin, amoxicillin, and tetracycline were high in Zhuanghe. The resistanc rates to levofloxacin and amoxicillin increased over time. Clarithromycin resistance was associated with male and iceA. The resistance of metronidazole was related to vacA. Levofloxacin resistance was concerned with cagA and slyD and amoxicillin resistance was concerned with iceA. While, the antibiotic resistance of *H. pylori* had nothing to do with the status of gastric disease.

## Background

A number of large randomized controlled trials have shown that the eradication of *Helicobacter pylori* (*H.pylori*) can reduce the occurrence of chronic atrophic gastritis and intestinal metaplasia [1] and decrease the risk of gastric cancer [2]. Therefore, the successful eradication of *H. pylori* is of great significance for the prevention and treatment of gastric diseases. The antibiotics that are commonly used for *H. pylori* eradication include clarithromycin, metronidazole, amoxicillin, and levofloxacin. In the past few decades, the resistance rate has continued to increase.

The antibiotic resistance of *H. pylori* exhibits regional differences. In Germany [3] and Brazil [4], the antibiotic resistance rate to clarithromycin was 28.7 and 23.2%, respectively, and it was only 7.3% in Ireland [5] and 0% in Malaysia [6]. The resistance rate to levofloxacin was 32.7% in Buenos Aires [7] and 18.2% in the Bogota-Colombia [8], as well as 5.8% in the Hebei Province of China [9]. As reported in the literature, the resistance rate to amoxicillin and tetracycline is generally low [7, 10, 11], but in Ireland and Vietnam, it was as high as 38.1% [5] and 23.8% [12], respectively.

There are many factors influencing the antibiotic resistance of *H. pylori*, including age and gender, as well as the host disease status and virulence factors carried by the pathogens. However, there is no consistent report on whether these factors are related to the antibiotic resistance of *H. pylori*. It is well known that the virulence factors secreted by *H. pylori* are closely related to different clinical outcomes in gastric diseases. Cytotoxin-associated gene A (*cagA*) is the most common virulence factor of *H. pylori*. Some studies have shown that the clarithromycin resistance rate is higher in patients with *cagA*^+^ than those with *cagA*^−^ [13], but others have demonstrated that the eradication rate in patients with *cagA*^*+*^ was significantly higher than those with *cagA*^−^ [14]. These studies showed that the influencing factors of *H. pylori* antibiotic resistance are different. Therefore, it is necessary to carry out further investigations in different areas.

Zhuanghe, Liaoning Province, a high-risk area for gastric cancer, is in northern China. The *H. pylori* infection rate for the local residents was more than 60%, and the risk of gastric cancer and precancerous diseases was increased for *H. pylori*–infected individuals [15]. In this study, we investigated the antibiotic resistance of *H. pylori* to metronidazole, levofloxacin, clarithromycin, amoxicillin, and tetracycline, aimed to provide guidance for the selection of medication, to improve the eradication effect, and to reduce the occurrence of gastric disease.

## Methods

### Patients

A total of 100 strains were collected from the primary cultures of 454 gastric mucosal tissues obtained during an endoscopic biopsy that were all from the Zhuanghe Gastric Diseases Screening Program, started in 1997 [15]. The gastric mucosal tissues were cultured for *H.pylori* during the 3 periods of 1998–1999, 2002–2004, 2016–2017 were 102, 242 and 110 cases. The number of primary cultured strains was 23, 50, and 27, respectively, so the total number of specimens in the 3 years was 100 strains. The patients were 54 males and 46 females, ranging from 24 to 87 years old (mean age: 50.74 ± 10.942 years). Pathological diagnosis was based on the updated Sydney Classification System, including 21 cases of superficial gastritis (GS), 35 cases of atrophic gastritis (GA), 32 cases of gastric erosion ulcer (GEU), 7 cases of atypical dysplasia (GD), and 5 cases of gastric cancer (GC). Patients were excluded if they had stomach surgery and if they were administered eradication treatment. The study was approved by the Ethics Committee of Chinese Medical University and all participants signed an informed consent.

### Antibiotic susceptibility testing

The susceptibility of *H. pylori* to metronidazole, levofloxacin, clarithromycin, amoxicillin, and tetracycline was tested using an Epsilometer test (E-test, bioMerieux, France). The methods of *H.pylori* culture and antibiotic susceptibility testing are all the same in different periods. The *H. pylori* that was in a logarithmic growth period was adjusted to a McFarland standard of 2.0 and smeared evenly with a sterile cotton swab (bioMerieux, France) on Mueller-Hinton agar (Oxoid, Basingstoke, UK), supplemented with 7% defibrinated sheep blood. The concentration range of the E-test strips was 0.016–256 μg/ml for metronidazole, amoxicillin, and tetracycline (bioMerieux, France); 0.016–256 μg/ml for clarithromycin; and 0.016–32 μg/ml for levofloxacin (Kangtai, China). Then, the plates were incubated at 37 °C for 48–72 h in a microaerophilic atmosphere with 10% O_2_, 5% CO_2_, and 85% N_2_. Thereafter, the minimum inhibitory concentration (MIC) for each antibiotic was determined after 48–72 h by two people independently. The MIC breakpoints were identified using the recommended Clinical and Laboratory Standards Institute (http://www.clsi.org/) of America. Strains were considered resistant with an MIC of ≥1 mg/ml for clarithromycin [16], levofloxacin [17], amoxicillin and tetracycline [18] and ≥ 8 mg/ml for metronidazole [4]. The *H. pylori* reference strain, ATCC 26695, was used for quality control.

### DNA extraction

Whole genomic DNA of *H. pylori* was extracted using a phenol-chloroform method [15]. In brief, 300 μl of TE buffer (10 Mm Tris-HCl [pH 8.3], 0.1 mmol/l EDTA), 100 μl of 10% SDS, and 10 μl of PK enzyme (20 mg/ml) were added to the bacterial precipitate and digested overnight in a water bath at 55 °C. The extracted DNA was dissolved in 50–100 μl of TE buffer and stored at − 20 °C.

### Polymerase chain reaction amplification of *H. pylori* virulence genes

*H. pylori* virulence factors *cagA*, *vacA* (s1, s2, m1a, m1b, m2, i1, and i2), *iceA*1, *iceA*2, *babA*2, *oipA*, *slyD*, *hrgA*, and *cagA*-EPIYA motif were amplified using polymerase chain reaction (PCR) [19–26]. The 25-μl amplification system included 2 mmol/L dNTP, 2.5 U Taq DNA polymerase, 10 × buffer (Takara, China), 10 pmol primer, and 1 μl of DNA template. The amplified products were identified using 2% agarose gel electrophoresis. Both positive and negative controls were performed in parallel for each PCR reaction.

### Statistical analysis

All statistical analysis was carried out using SPSS V.18.0 software for Windows (SPSS Inc., Chicago, IL, USA). A binary logistic regression model was used to calculate the odds ratios (OR) and 95% confidence interval (CI). A *P* value of < 0.05 was defined as significant.

## Results

### The characteristics of *H. pylori* antibiotic resistance

**Fig. 1:**
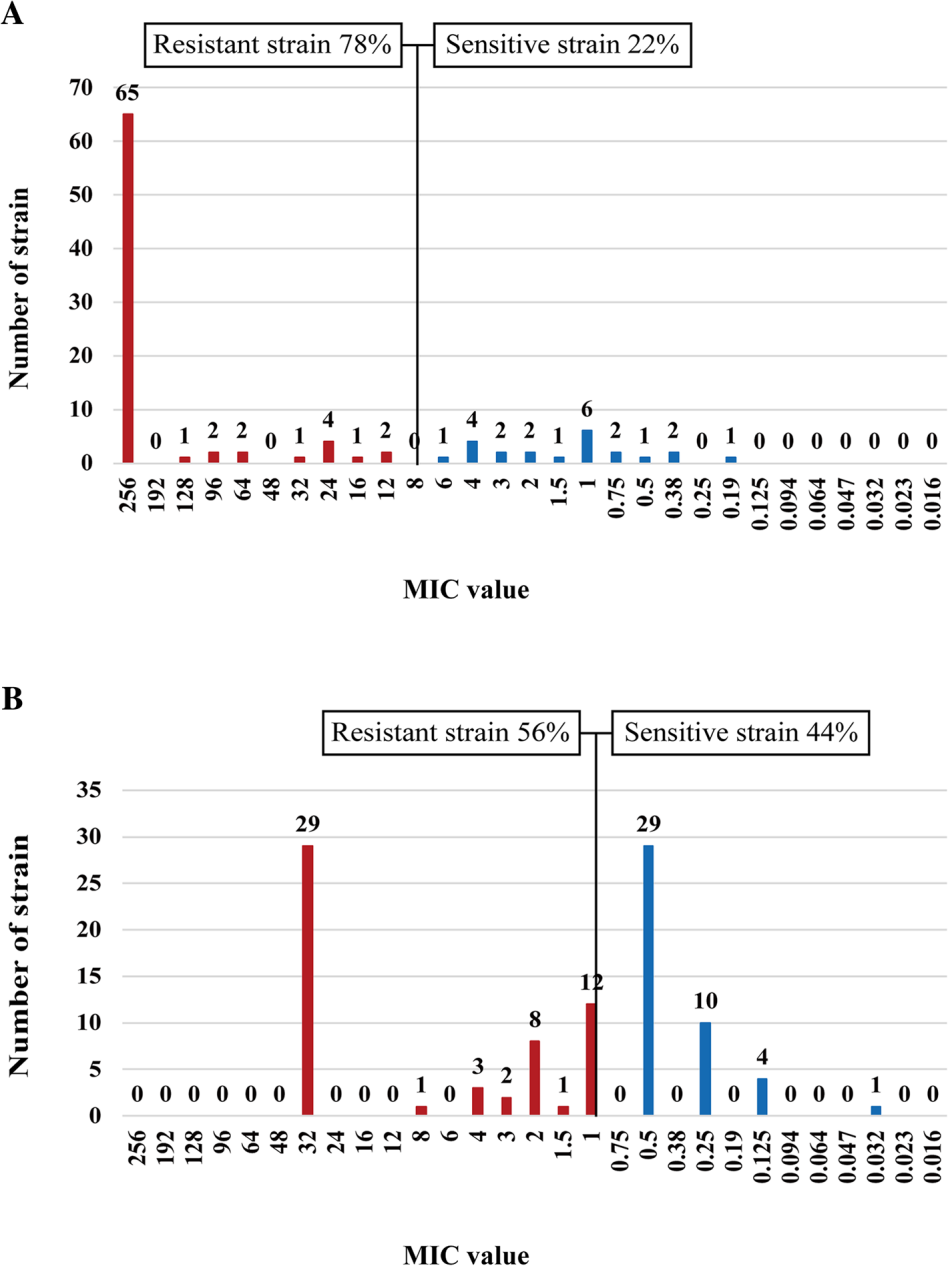
Distribution of the antibiotic (**a** for metronidazole, **b** forlevofloxacin), MIC values for the *H. pylori* isolates in Zhuanghe

**Fig. 2:**
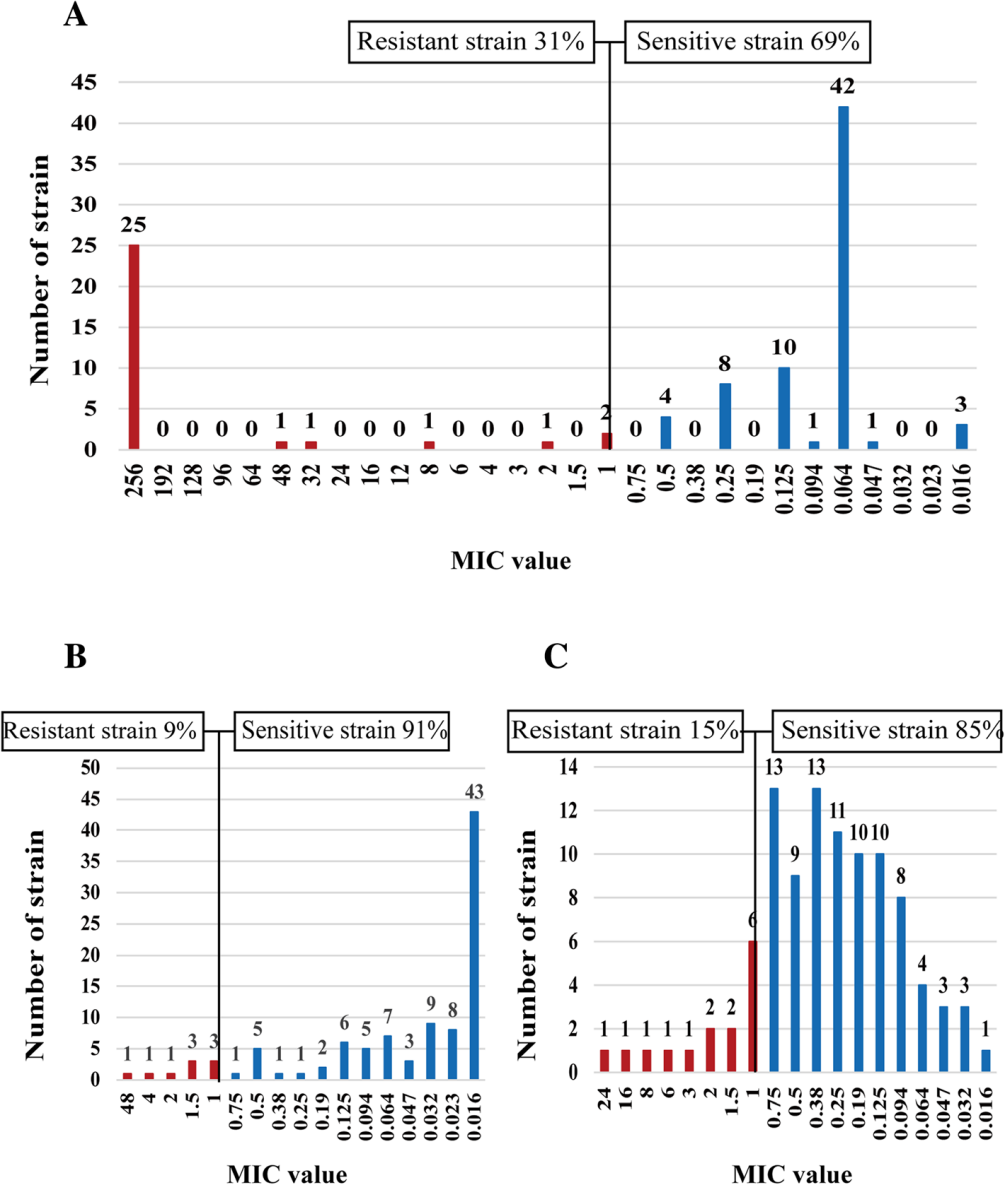
Distribution of the antibiotic (**a** for clarithromycin, **b** for amoxicillin, and **c** for tetracycline). MIC values for the *H. pylori* isolates in Zhuanghe

First, we detected the drug resistance of 100 *H. pylori* strains to the five previously mentioned antibiotics using E-test. The results showed that the total resistance rates of *H. pylori* to metronidazole, levofloxacin, clarithromycin, amoxicillin, and tetracycline were 78.0% (78/100), 56.0% (56/100), 31.0% (31/100), 9.0% (9/100), and 15.0% (15/100), respectively (Figs. 1 and 2). Further analysis revealed that *H. pylori* resistance occurred at high MIC levels. In the metronidazole-resistant strains, 89.7% (70/78) of the MIC values were more than 64 μg/ml and 83.3% (65/78) exceeded 256 μg/ml (Fig. 1a). In the levofloxacin-resistant strains, 51.8% (29/56) of the MIC values were 32 μg/ml (Fig. 1b). Among the strains resistant to clarithromycin, 80.6% (25/31) of the MIC values were above 256 μg/ml (Fig. 2a). If stratified by gender and age, we found that the clarithromycin resistance rate was significantly higher for males than females (44.4% vs 15.2%, respectively; OR: 4.457, 95%CI: 1.694–11.724; *P* = 0.002). There was no significant difference for the resistance rates of the four other antibiotics by gender. Further, no significant difference was demonstrated in the resistance rates for the five antibiotics by age (Table 1).

**Table 1:** The Characteristic of antibiotic resistance of *H. pylori* isolates in Zhuanghe

Characteristic	N	MNZ		CAM		LVX		AMX		TCN	
		OR(95%CI)	*P* value	OR(95%CI)	*P* value	OR(95%CI)	*P* value	OR(95%CI)	*P* value	OR(95%CI)	*P* value
Age											
≤ 60 vs > 60	17 vs 83	0.420 (0.088–1.996)	0.275	*0.581 (0.198–1.705)*	*0.323*	1.160 (0.407–3.305)	0.780	1.778 (0.207–15.236)	0.6	0.824 (0.205–3.306)	0.784
Gender											
Female vs male	46 vs 54	1.553 (0.600–4.020)	0.364	**4.457 (1.694–11.724)**	**0.002**	1.571 (0.709–3.483)	0.266	1.745 (0.410–7.422)	0.451	2.619 (0.770–8.903)	0.123
Clinical outcome											
GEU vs NGEU	32 vs 63	1.006 (0.318–3.182)	0.992	*0.857 (0.325–2.261)*	*0.756*	0.613 (0.242–1.553)	0.302	1.180 (0.252–5.537)	0.834	1.886 (0.504–7.056)	0.346
GA vs GS	36 vs 20	4.356 (0.854–22.223)	0.077	*0.258 (0.058–1.138)*	*0.073*	0.608 (0.202–1.832)	0.377	0.301 (0.032–2.808)	0.292	1.567 (0.342–7.180)	0.563
GEU vs GS	32 vs 20	1.683 (0.496–5.709)	0.403	*0.632 (0.201–1.982)*	*0.431*	1.694 (0.586–4.898)	0.33	0.534 (0.101–2.816)	0.46	0.74 (0.162–3.417)	0.704
GEU vs GA	32 vs 36	0.463 (0.063–3.395)	0.449	*3.182 (0.563–17.972)*	*0.19*	2.197 (0.628–7.691)	0.218	0.966 (0.074–12.669)	0.979	0.214 (0.034–1.368)	0.103
Year											
1998 to 2017	100	1.276 (0.638–2.551)	0.491	*1.543 (0.823–2.892)*	*0.176*	**2.089 (1.142–3.821)**	**0.017**	**5.035 (1.327–19.105)**	**0.018**	1.161 (0.540–2.499)	0.702
Virulence factors											
cagA- vs cagA+	13 vs 87	1.509 (0.409–5.561)	0.537	*2.192 (0.427–11.235)*	*0.347*	**5.133 (1.297–20.319)**	**0.02**	0	0.999	0	0.999
babA2- vs babA2+	18 vs 82	1.374 (0.423–4.456)	0.597	*0.822 (0.257–2.627)*	*0.741*	0.789 (0.276–2.303)	0.676	0.854 (0.158–4.634)	0.855	1.606 (0.316–8.159)	0.568
hrgA- vs hrgA+	43 vs 57	0.518 (0.188–1.431)	0.205	*0.419 (0.166–1.059)*	*0.066*	0.708 (0.315–1.591)	0.403	1.839 (0.458–7.385)	0.391	1.749 (0.569–5.376)	0.329
slyD- vs slyD+	54 vs 46	0.519 (0.196–1.376)	0.187	*0.585 (0.233–1.470)*	*0.254*	**0.334 (0.146–0.764)**	**0.009**	0.144 (0.017–1.205)	0.074	0.883 (0.282–2.765)	0.831
opiA- vs opiA+	33 vs 67	0.435 (0.141–1.340)	0.147	*1.163 (0.424–3.193)*	*0.769*	0.501 (0.203–1.236)	0.133	0.404 (0.100–1.632)	0.203	0.557 (0.179–1.731)	0.312
vacAi1- vs vacAi1+	36 vs 64	0.490 (0.161–1.494)	0.21	*0.448 (0.174–1.154)*	*0.096*	0.891 (0.380–2.092)	0.791	0.427 (0.106–1.720)	0.231	0.589 (0.190–1.824)	0.359
vacAs1m1m2 vs vacAs1m1/m2	42 vs 40	**0.287 (0.096–0.863)**	**0.026**	*0.763 (0.287–2.027)*	*0.588*	0.749 (0.311–1.804)	0.52	0.434 (0.078–2.420)	0.341	0.758 (0.215–2.667)	0.666
iceA-- vs iceA−+	18 vs 21	0.505 (0.071–3.612)	0.496	*1.476 (0.369–5.902)*	*0.582*	0.572 (0.135–2.415)	0.447	0.489 (0.090–2.649)	0.406	1.096 (0.222–5.418)	0.91
iceA-- vs iceA++	18 vs 61	0.317 (0.065–1.557)	0.157	*0.310 (0.094–1.020)*	*0.054*	0.428 (0.134–1.367)	0.152	**0.051 (0.005–0.481)**	**0.009**	0.450 (0.106–1.900)	0.277
iceA− + vs iceA++	21 vs 61	0.474 (0.122–1.847)	0.282	**0.234 (0.071–0.775)**	**0.017**	0.812 (0.294–2.240)	0.688	0.101 (0.009–1.086)	0.059	0.388 (0.099–1.527)	0.176

In addition, among the 100 strains of *H. pylori*, 39 (39%) had single-drug resistance, 23 (23%) had double-drug resistance, and 20 (20%) had triple-drug resistance; meanwhile, 6 (6%) were resistant to four drugs, and 4 (4%) were resistant to all five antibiotics (Table 2). Only 8 (8%) strains were found to be sensitive to all five antibiotics. As for the multidrug-resistant strains, most were double or triple resistant. The highest proportion of a double-drug resistant pattern was metronidazole plus levofloxacin, accounting for 73.9%. The highest proportion of a triple-drug resistance pattern was the combination of metronidazole, levofloxacin, and clarithromycin, accounting for 55%. Similarly, after stratifying by gender and age, the results showed that the multidrug resistance rate was higher for males than females (75.5% vs 37.2%, respectively; OR: 5.203, 95%CI: 2.120–12.771, *P* < 0.001, Table 1). However, there was no significant difference between the single and multidrug resistance rates based on age.

**Table 2:** Distribution of resistance patterns among *H. pylori* strains

Susceptibility test results	N
Sensitive to all ABs	8
Single	39
MNZ	26
LVX	9
CAM	3
AMX	0
TCN	1
Double	23
MNZ + LVX	17
MNZ + CAM	5
LVX + CAM	1
Triple	20
MNZ + LVX + CAM	11
MNZ + LVX + TCN	6
MNZ + LVX + AMX	2
MNZ + CAM + AMX	1
Quadruple	6
MNZ + LVX + CAM + AMX	2
MNZ + LVX + CAM + TCN	4
Resistance to all ABs	4

Abbreviations: *AB* antibiotic

### The changing profile of *H. pylori* antibiotic resistance during the past three decades

**Fig. 3:**
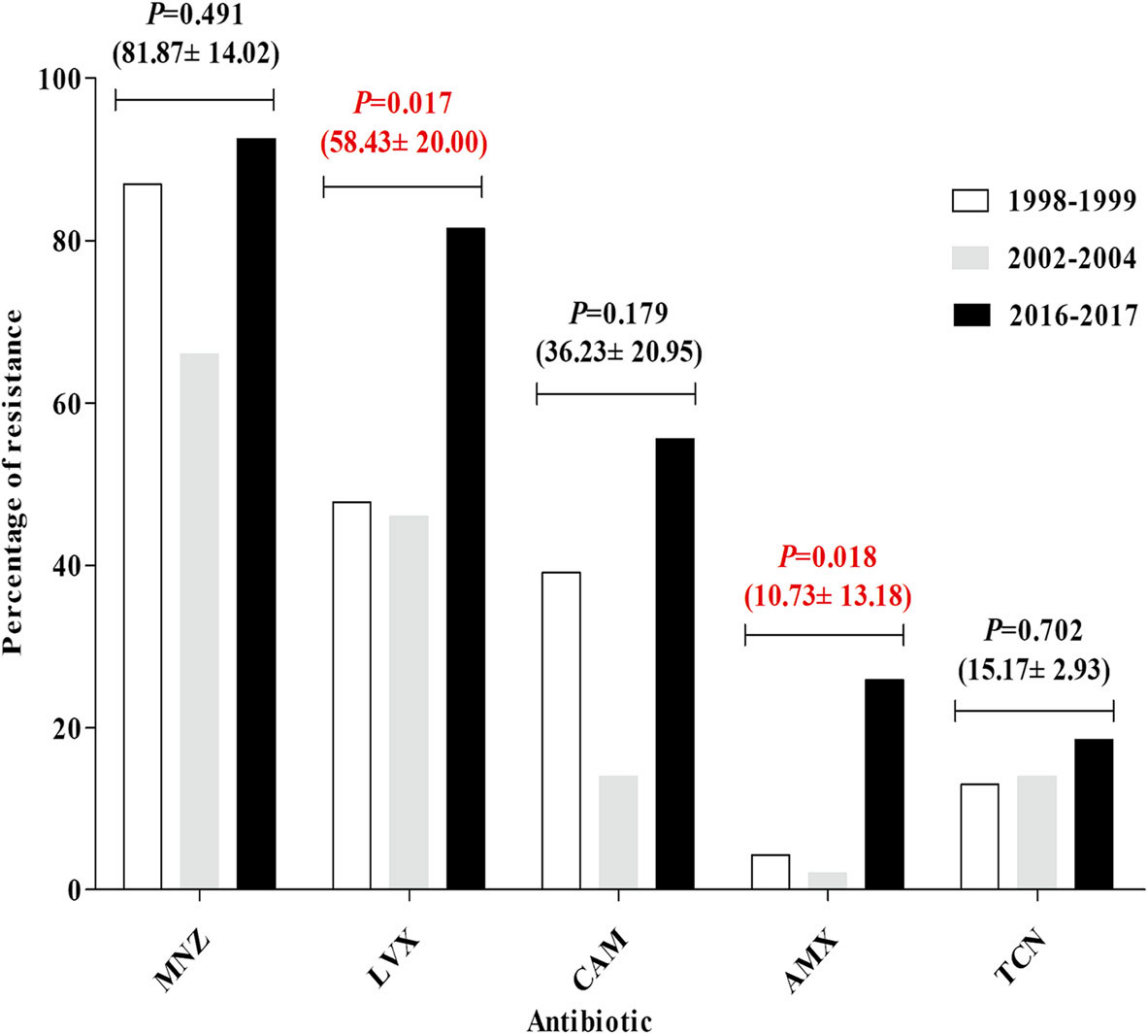
The changing profile for *H. pylori* antibiotic resistance during the three decades

In this study, we compared the changing profile of *H. pylori* resistance in the Zhuanghe area during three decades from 1998 to 2017 (Fig. 3). During the three periods, 1998–1999, 2002–2004 and 2016–2017, the resistance rate to certain antibiotics was as follows: metronidazole was 87.0, 66.0 and 92.6%, respectively; levofloxacin was 47.8, 46.0 and 81.5%, respectively; clarithromycin was 39.1, 14.0 and 55.6%, respectively; amoxicillin was 4.3, 2.0 and 25.9%, respectively; and tetracycline was 13.0, 14.0 and 18.5%, respectively. Adjusting for age and gender, we found that the resistance rates to metronidazole, clarithromycin, and tetracycline were not significant over the three decades (Table 1), but the resistant rates to levofloxacin and amoxicillin increased (OR: 2.089, 95%CI: 1.142–3.821, *P* = 0.017, respectively; OR: 5.035, 95%CI: 1.327–19.105, *P* = 0.018, respectively). In addition, we analyzed the changing profile for the single-drug or multidrug resistance rates over the three decades (Table 1) that showed that the rate of single-drug resistance decreased (OR: 0.411, 95%CI: 0.205–0.824, *P* = 0.012), but the multidrug resistance increased (OR: 2.059, 95%CI: 1.090–3.889, *P* = 0.026).

### The antibiotic resistance of *H. pylori* and host disease state

Concerning the relationship between *H. pylori* antibiotic resistance and host disease status, we found that after adjusting for age and gender, there was no significant difference in the antibiotic resistance for the GS, GA, and GEU groups or the GEU and non-gastric erosion ulcer (NGEU) groups (Table 1).

### The antibiotic resistance of *H. pylori* and virulence factor

The virulence factors *cagA*, *vacA* (s1, m1, m2, and 1), *iceA*1, *iceA*2, *babA*2, *oipA*, *slyD*, and *hrgA* and their correlations with the five types of antibiotic resistance were calculated by a binary logistic regression analysis (Table 1). The results showed that the resistance rate to metronidazole was lower in the *vacA*s1m1/m2 group than the *vacA*s1m1m2 group (65% vs 85.7%, respectively; OR: 0.287, 95%CI: 0.096–0.863, *P* = 0.026) after adjusting for age and gender. As for the resistance rate to levofloxacin, it was higher in the *cagA* ^+^ group than the *cagA*^−^ group (60.9% vs 23.1%, respectively; OR: 5.133, 95% CI: 1.297–20.319, *P* = 0.020) but was lower in the *slyD*^+^ group than the *slyD*^−^ group (41.4% vs 68.5%, respectively; OR: 0.334, 95%CI: 0.146–0.764, *P* = 0.009). A lower resistance rate to clarithromycin was observed in the *iceA*^++^ group than the *iceA*^- +^ group (19.7% vs 52.4%, respectively; OR: 0.234, 95%CI: 0.071–0.775, *P* = 0.017). As for amoxicillin, the *iceA*^++^ group had a lower resistance rate than the *iceA*^−−^ group (1.6% vs 27.8%, respectively; OR: 0.051, 95%CI: 0.005–0.481, *P* = 0.009). There was no significant difference in the relationship between the other *H. pylori* virulence factors such as *vacA*, *babA*, *oipA*, and *hrgA* and antibiotic resistance (*P* > 0.05). In addition, to detect the EPIYA motif classifications for the *cagA*^+^ (*n* = 87) group, we found that it was mainly composed of East Asian type EPIYA-ABD (*n* = 79), western type EPIYA-ABC (*n* = 2), mixed type EPIYA-ABCD (*n* = 5), and one EPIYA-ACD. Because of the small number of cases in the different groups, the statistical relationships for antibiotic resistance cannot be analyzed.

## Discussions

Antibiotic resistance is the main reason for failure to eradicate an *H. pylori* infection. In this study, 100 *H. pylori* strains from Zhuanghe, an area with a high incidence of GC, were included to investigate the antibiotic resistance, to explore the trend over time, and to analyze the factors affecting antibiotic resistance. This study revealed the antibiotic resistance of *H. pylori* to five antibiotics in an area with a high incidence of GC and provided guidance for the treatment of *H. pylori*.

Our study demonstrated that the total resistance rates of *H. pylori* to metronidazole, levofloxacin, and clarithromycin were 78, 56, and 31%, respectively, with high MIC levels; however, the resistance rates to amoxicillin and tetracycline were low, and the multidrug resistance rate was as high as 53%. As compared with low-risk areas for GC in China [27–29], neighboring countries [30–33], and other non-neighboring countries [6, 11, 34, 35], the antibiotic resistance was generally higher in the Zhuanghe area. The high antibiotic resistance increased the risk of *H. pylori* transmission among the population and caused a widespread and persistent high infection rate of *H. pylori* in the population; moreover, it also could increase the continued colonization of the stomach in hosts and increase the inflammation of and damage to the gastric mucosa, which may lead to an increased risk of GC.

According to previous studies, *H. pylori* antibiotic resistance has significantly changed over time [8, 33, 36–38]. For example, in Korea, the resistance rate to clarithromycin increased from 11 to 60% from 2009 to 2014 [39, 40]. In Singapore, the resistance rate to metronidazole increased from 24.8 to 48.2% from 2000 to 2014 [38]. Our results showed that the resistance rates to clarithromycin and metronidazole maintained a stable yet high level and that tetracycline was at a steady yet low level in 1998–2017. The resistance rates to levofloxacin and amoxicillin, as well as multidrug resistance, have demonstrated an increasing trend. Our study showed that the two antibiotics, clarithromycin and metronidazole, were not suitable for eradication therapy in this area. However, tetracycline can be used to eradicate *H. pylori* but has a shortcoming because of adverse reactions, so the time and dose should be strictly controlled. Developing alternative drugs to help with eradication is also a future goal.

In this study, we found that the resistance rate to clarithromycin for males was significantly higher than females. *Boyanova* [41] also had similar conclusions in females and males. In addition, we found males are more prone to multidrug resistance than females. The possible causes of this phenomenon was that macrolide antibiotics were commonly used for the treatment of respiratory diseases and respiratory infections related to smoking and other factors mostly occurred in men that might lead to antibiotic resistance for males with a clarithromycin-based eradication treatment for *H. pylori*. Previous studies of our group showed that the incidence and mortality of males with GC in the Zhuanghe area were higher than those of females by 2.8 times and 2.3 times, respectively [42]. Thus, the high antibiotic resistance of *H. pylori* for males might also be one risk factor for GC in addition to environmental exposure factors such as diet, smoking, drinking, and occupation.

In addition, there have been different reports on whether the gastric disease status is related to the antibiotic resistance of *H. pylori*. A study involving 2751 patients in France showed that the successful eradication rate of *H. pylori* in patients with a duodenal ulcer was higher than that of patients with a non-ulcer-related dyspepsia [43]. However, some medical literature indicated that these diseases are not related to antibiotic resistance [30, 41, 44–47]. In the present study, we found that the *H. pylori* resistance rate was not associated with gastric disease. However, while analyzing the reason, on the one hand, it may be geographical differences. On the other hand, it may be because of the small number of cases included in this study.

Some virulence factors carried by *H. pylori* may also affect the clinical outcomes of eradication [48–50]. As compared with single- and multi-strain infections, we found that the resistance rate of metronidazole to *vacA*m1 or *vacA*m2 was lower than that of a mixed infection with *vacA*m1m2, to *iceA*1 or *iceA*2 alone higher than that of mixed infection with *iceA*1*A*2. The resistance rate to amoxicillin in *iceA*^*−−*^ was higher than that of a mixed infection. Additionally, the resistance rate to levofloxacin was higher in the *cagA* ^*+*^ group than the *cagA*^*−*^ one. The resistance rate to levofloxacin was higher in the *slyD*^*−*^ group than the *slyD*^*+*^ one. *CagA*^*+*^ or mixed *vacA*s1m1m2 infection was associated with antibiotic resistance. Patients infected with such strains had more difficulty achieving eradication of *H. pylori,* and continuous colonization was more likely to induce precancerous lesions and GC. Therefore, decreasing the incidence of GC could be achieved so long as the emergence of antibiotic resistance is avoided during the administration of effective eradication treatment. In addition, a mixed *iceA* or *slyD*^*+*^ strain would be easy to eradicate according to the present study results. However, in some reports both *iceA*1 and *iceA*2 infections were unrelated to the eradication outcomes [51, 52]. *SlyD* was a new virulence factor associated with gastric disease [53]. To our knowledge, until now, there was no report on the relationship between *slyD* and antibiotic resistance. Therefore, the relationship between these two virulence factors and bacterial resistance needs to be further explored.

This study had the following limitations: 1. Since there exists dominant genotype of *H.pylori* virulence factors eg. *cagA* positive, *vacA* s1 m1, and *iceA*1 positive in this area [15], therefore, the relationship between virulence factors and antibiotic resistance needs to be further investigated. 2. The obtained conclusions in the present study were based in a number of only 100 strains, the factors affecting the antibiotic resistance still needs to further verification in a large samples. 3. Other factors such as host CYP2C19, MDR, and IL-1β gene polymorphism can also influence *H. pylori* eradication. However, there is a lack of relevant information in our study, so we could not evaluate it.

## Conclusions

In conclusion, our study evaluated the antibiotic resistance and influencing factors of *H. pylori* in Zhuanghe. The total resistance rates to metronidazole, levofloxacin, clarithromycin, amoxicillin, and tetracycline were high. The resistance rates to levofloxacin and amoxicillin increased over time. Clarithromycin resistance was associated with male and iceA. The resistance of metronidazole was related to *vacA*. Levofloxacin resistance was concerned with *cagA* and *slyD* and amoxicillin resistance was concerned with *iceA*. While, the antibiotic resistance of *H. pylori* had nothing to do with the status of gastric disease. Per this study, clarithromycin-based triple therapy is no longer applicable in this area; metronidazole and levofloxacin should be used with high vigilance; and amoxicillin and tetracycline can be used as candidates for antibiotic treatment. Virulence factors including *cagA*, *vacA*, *iceA,* and *slyD* can be used to predict antibiotic resistance. Our study is beneficial to the selection of the *H. pylori* eradication program that is conducive to reducing the infection rate, thus helping in the prevention and treatment of GC and related gastric diseases.

## Data Availability

All data generated or analysed during this study are included in this published article.

## Abbreviations

AMX: Amoxicillin
CAM: Calrithromycin
GA: Atrophic gastritis
GC: Gastric cancer
GD: Atypical dysplasia
GEU: Gastric erosion ulcer
GS: Superficial gastritis
LVX: Levofloxacin
MIC: Minimum inhibitory concentration
MNZ: Metronidazole
NGEU: Non-gastric erosion ulcer (NGEU)
TCN: Tetracycline
